# Previous antibiotic therapy as independent risk factor for the presence of vancomycin-resistant enterococci in surgical inpatients. Results from a matched case-control study

**DOI:** 10.1186/s12879-023-08238-4

**Published:** 2023-05-02

**Authors:** Philip MacKenzie, Jacqueline Färber, Marius Post, Torben Esser, Lukas Bechmann, Siegfried Kropf, Roland Croner, Gernot Geginat

**Affiliations:** 1 Department of Medical Microbiology and Infection Control, Otto-von-Guericke University Magdeburg, Magdeburg, Germany; 2 Institute for Biometry and Medical Informatics, Otto-von-Guericke University, Magdeburg, Germany; 3 Department of General, Visceral, Vascular and Transplant Surgery, Otto-von-Guericke University Magdeburg, Magdeburg, Germany; 4 Institut für medizinische Mikrobiologie und Krankenhaushygiene, Otto-von-Guericke University, Leipziger Straße 44, 39120 Magdeburg, Germany

**Keywords:** Vancomycin-resistant enterococci, Risk-factors, Case control study, Surgical patients

## Abstract

**Background:**

Investigation of risk factors for the presence of vancomycin-resistant enterococci (VRE) in inpatients on surgical wards and associated intensive care units of a German tertiary care hospital.

**Methods:**

A single-centre retrospective matched case-control study was performed with surgical inpatients admitted between July 2013 and December 2016. Patients with in-hospital detection of VRE later than 48 h after admission were included and comprised 116 VRE-positive cases and 116 VRE-negative matched controls. VRE isolates of cases were typed by multi-locus sequence typing.

**Results:**

ST117 was identified as the dominant VRE sequence type. Next to length of stay in hospital or on an intensive care unit and previous dialysis the case-control study revealed previous antibiotic therapy as a risk factor for the in-hospital detection of VRE. The antibiotics piperacillin/tazobactam, meropenem, and vancomycin were associated with the highest risks. After taking into account length of stay in hospital as possible confounder other potential contact-related risk factors such as previous sonography, radiology, central venous catheter, and endoscopy were not significant.

**Conclusions:**

Previous dialysis and previous antibiotic therapy were identified as independent risk factors for the presence of VRE in surgical inpatients.

## Background

Vancomycin-resistant *Enterococcus faecium* (VRE) are common nosocomial pathogens in Germany [1]. Risk factors that are associated with colonisation and infection of inpatients by VRE include previous antibiotic therapy, previous hospitalisation, and invasive interventions [2–4]. After colonization with VRE some patients have an enhanced risk for developing an invasive VRE infection, in particular immunocompromised patients such as haematological-oncological patients, liver-transplanted patients, patients awaiting liver transplantation, patients on renal dialysis, and neonates. VRE bacteraemia has been associated with increased mortality compared to vancomycin-sensitive enterococcal bacteraemia [3], especially in neutropenic and haematological-oncological patients [5–7].

The ability of enterococci to survive on inanimate surfaces for extended periods augments in-hospital transmission of VRE [8]. Surfaces in rooms that accommodate VRE-colonised patients are rapidly contaminated with VRE and patient rooms previously occupied by a VRE-colonised inpatient have been identified as a risk factor for the in-hospital acquisition of VRE [9].

The aim of the present retrospective study was to identify risk factors associated with the detection of VRE colonisation during the first surge of VRE on surgical wards and attached intensive care units (ICU) in our hospital between 2013 and 2016. For this purpose, a case-control study was initiated with surgical and ICU inpatients who acquired VRE during this period.

## Methods

### Typing of VRE

For rectal screening of patients ESwab™ (COPAN Diagnostics INC., USA) was used. The liquid medium was inoculated on a chromogenic selective agar medium (ChromID VRE, bioMérieux, France). After incubation for 48 h at 35 ± 1 °C presumptive VRE colonies were confirmed by MALDI-TOF MS (VITEK® MS, bioMérieux, France) followed by multiplex polymerase chain reaction (PCR) artus® VanR QS-RGQ Kit (Qiagen, Hilden, Germany). Multilocus sequence typing (MLST) was performed as described [10], targeting seven housekeeping genes of *E. faecium* (atpA, ddl, gdh, purK, gyd, pstS adk). For editing, alignment, and phylogenetic analysis of sequences the MEGA 6.0 software package was used.

### Retrospective case-control study

A checklist with known risk factors (stay and duration on ICU, stay and duration in hospital, dialysis, antibiotic therapy, central venous catheter, sonography, radiologic imaging, and endoscopy) associated with nosocomial colonisation or infection of patients was developed and used to retrospectively check patient records. In the case group only risk factors present before the first detection of VRE were recorded. In the control group all risks factors present before the last VRE-negative swab were recorded. The duration of antibiotic therapy was recorded as days of antibiotic therapy (DOT) for each antibiotic used. Invasive and non-invasive clinical procedures were recorded qualitatively only. If data were incomplete or missing, patients were excluded from the study.

The implementation of the present case-control study was reviewed and approved by the Ethics Committee of the University Hospital Magdeburg (file number 126/17, approval date 24.08.2017). The study was performed in accordance with the Declaration of Helsinki. The data processing was based on pseudonymized patient data and did not include any experiments involving human participants (including the use of tissue samples). The requirement for informed consent was waived by the Ethics Committee of the University Hospital Magdeburg because of the retrospective nature of the study.

All patients of the included surgical wards and surgical ICUs between July 2013 and December 2016 with a positive VRE culture detected later than 48 h after admission were included in the case group. The included surgical wards mainly comprised general and vascular surgery. At the time of the study a routine VRE-screening upon admission of patients was not implemented and was not required for the inclusion of cases.

If multiple positive samples were available for one patient, only the first sample was recorded. Applying these criteria 239 patients were included in the primary study group. In order to create a homogeneous case group and thus enable comparability of the patients, we excluded all patients, who during the current stay had been on a non-surgical ward of the hospital for more than two days before admission to one of the above-mentioned wards. Inpatients from gynecology and obstetrics were excluded because the clinic is located in another part of the city. In addition, patients younger than 18 years were excluded, as paediatric patients differ from adults in disease patterns and comorbidities as well as medical care and are thus difficult to compare with adult patients. After excluding patients according to these criteria, we obtained a case group with 118 patients out of the original 239 patients. One patient was excluded as files could not be retrieved and a further patient was excluded because records for the matched partner in the control group could not be retrieved. Thus, the final sample size was n = 116 (Table 1).

**Table 1:** Selection of patients for the case and control groups

	cases	controls
patients fullfilling criteria for inclusion^1^	239	8078
patients after application of criteria for exclusion^2^	118	1424
after matching	118	118
exclusion due to incomplete data of a case	1	1
exclusion due to incomplete data of a control	1	1
final matched groups	116	116

^1^Inclusion criteria: inpatient between second half of 2013 to 2016 on a surgical ward or a surgical intensiv care unit and detection of VRE later than 48 h after admission (cases) or VRE-negative (controls).

^2^Exclusion criteria: inpatient on a non-surgical ward or in the gynaecology and obstetrics clinic for more than 2 days before admission to one of the included surgical wards, or younger than 18 years.

Possible patients to be included in the control group primarily comprised all patients with at least one VRE-negative culture test later than 48 h after admission in the study period (n = 8078). In order to ensure the quality of rectal sampling, VRE-negative swabs were only considered truly negative if there was growth of typical enteric bacteria on sheep blood agar. Patients with a subsequent positive VRE culture were excluded. As in the case group, patients who stayed on a non-surgical ward prior to admission to one of the included wards were excluded in order to create homogeneity and comparability of the groups. Before matching the list of possible controls comprised 1424 patients.

Matching of the case group with the control group was performed in a ratio of 1:1 based on the following criteria: age ± 2 years, identical sex, sample collection period (sample collection in the periods 2013/14 and 2015/16, respectively). We did not include more than one control per case because limited personal resources. If the total number of cases and controls that can be included is limited, a 1:1 ratio yields the best statistical power and avoids unwanted selection effects in the case group [11].

### Statistics

Since cases and controls are unevenly distributed over the study period and the number of controls exceeded the number of cases by far, matched pairs of cases and controls were formed for further analyses using the FUZZY extension command in SPSS with matching criteria as defined above. Matching by ward was not carried out, as this was one of the possible target variables and patients were often transferred to several wards during their hospital stay.

Testing for significant differences between cases and controls was done primarily with tests for dependent samples accounting for pair formation [12]. However, since the pairs are independent individuals, sensitivity analyses were performed with tests for independent samples. As length of stay at hospital can be expected to be a confounder, the unadjusted comparisons between both groups have been complemented by comparisons with length of stay as covariable (except for length of stay at ICU which is highly correlated with this covariable). Depending on the scale of the variables, we used McNemar/Bowker tests or Wilcoxon matched-pairs signed-rank tests for the unadjusted paired comparisons, chi-squared test or Mann-Whitney U tests for unadjusted unpaired comparisons, generalised mixed linear models or mixed linear models for adjusted paired comparisons and logistic regression or analysis of covariance for adjusted unpaired comparisons. In order to present comparable effect measures for all factors, odds ratios were derived from logistic regression analyses. For the metrical factors, here measured as day counts, the odds ratios assess the risk increase for one additional day (of stay or under antiobiotic therapy).

The adjusted paired analyses for a pre-specified selection of six variables (including dialysis, antibiotic therapy, central venous catheter, sonography, radiologic imaging, and endoscopy before the detection of a VRE colonisation/infection) will be assessed in a confirmatory sense using Bonferroni-corrected significance thresholds of 0.05/6 for the two-sided p-values. Furthermore, if hereby antibiotic therapy is significant, then 18 subclasses of antibiotics will be assessed with significance thresholds of 0.05/(6*18). All other tests will be carried out as exploratory analyses at an unadjusted error level of 0.05. Associations between MSLT type and risk factors in the case group were analysed with chi square tests using the exact test distribution. All statistical analyses have been performed with IBM® SPSS® Statistics, version 25.

## Results

In order to define specific risk factors for the in-hospital acquisition of VRE as possible targets for an infection control intervention a retrospective case-control study was performed for the period from July 2013 to December 2016. For the case-control study 116 VRE cases and matched controls were selected using the inclusion and exclusion criteria described in the materials and methods section (Table 1).

The pairwise univariate analysis identified length of stay in hospital, length of stay on an ICU, dialysis, endoscopy, sonography, radiology (x-ray imaging, computed tomography scan, Magnetic resonance imaging), central venous catheter, and previous antibiotic therapy as risk factors. Generally, the results of analyses with consideration of matched pairs gave very similar results to the analyses as independent samples. Table 2 shows the results as odds ratios from logistic regression analyses for unpaired samples including test results.

**Table 2:** Risk factors associated with in-hospital detection of VRE in primary and secondary analysis in logistic regression analyses

risk factor	cases	controls	uni-variate^1^		multi-variate^,2^	
			OR^3^ (95% CI^3^) p-value		OR (95% CI) p-value	
LOS^3^ hospital, median (IQR)	15 (9–28)	7 (3, 12)	1.054 (1.028–1.081) **p ≤ 0.0001**			
LOS ICU^3^, median (IQR)	4 (1-10.5)	1 (0, 2)	1.114 (1.056–1.175) **p ≤ 0.0001**			
dialysis, n (%)	28 (24.1)	8 (6.9)	4.295 (1.864–9.897) **p ≤ 0.0005**		3.309 (1.387–7.897) **p ≤ 0.0082**	
CVC^3^, n (%)	104 (89.7)	89 (76.7)	2.629 (1.259–5.492) **p ≤ 0.0167**		1.663 (0.769–3.597) p ≤ 0.3443	
endoscopy, n (%)	55 (47.4)	34 (29.3)	1.802 (1.014–3.202) **p ≤ 0.0065**		1.403 (0.761–2.585) p ≤ 0.0821	
sonography, n (%)	90 (77.6)	64 (55.2)	2.812 (1.591–4.971) **p ≤ 0.0009**		2.169 (1.194–3.940) p ≤ 0.0145	
radiology (X-ray, CT, MRT), n (%)	102 (12.1)	78 (67.2)	3.549 (1.798–7.006) **p ≤ 0.0004**		2.327 (1.136–4.769) p ≤ 0.0623	
antibiotics DOT, median (IQR)	16 (10–28)	4 (2, 11.5)	1.082 (1.052–1.112) **p ≤ 0.0001**		1.069 (1.037–1.102) **p ≤ 0.0001**	
	mean (IQR)		OR (95% CI) p-value			
P/T^3^ DOT	4.7 (0-7.5)	2.2 (0–2)	1.139 (1.067–1.216) **0.0001**		1.116 (1.043–1.195) 0.0006	
A/S^3^ DOT	0.7 (0–0)	0.2 (0–0)	1.131(0.962–1.329) 0.1465		1.132 (0.945–1.356) 0.2069	
meropenem DOT	3.9 (0-7.5)	0.8 (0–0)	1.198 (1.103–1.301) **0.0001**		1.154 (1.058–1.258) 0.0005	
imipenem DOT	0.3 (0–0)	0.3 (0–0)	1.013 (0.879–1.169) 0,6406		0.940 (0.810–1.093) 0.4870	
CEPH^3^ 2nd gen DOT	0.8 (0–1)	1.1 (0–1)	0.926 (0.798–1.074) **0.0047**		0.953 (0.822–1.104) 0.4427	
CEPH^3^ 3rd gen DOT	0.7 (0–0)	0.4 (0–0)	1.067 (0.923–1.233) 0.1793		1.069 (0.921–1.241) 0.3129	
ciprofloxacin DOT	2.1 (0–2)	0.7 (0–0)	1.147 (1.039–1.268) **0.0022**		1.114 (1.002–1.238) 0.0502	
levofloxacin DOT	0.2 (0–0)	0.2 (0–0)	1.040 (0.813–1.329) 0,8359		0.931 (0.697–1.245) 0.6474	
metronidazole DOT	2.1 (0–3)	1.6 (0-1.5)	1.052(0.967–1.144) 0.5788		1.063 (0.975–1.160) 0.1267	
vancomycin DOT	2.7 (0–3)	0.5 (0–0)	1.215(1.097–1.346) **0.0001**		1.150(1.036–1.276) 0.0031	
macrolids DOT	0.9(0–0)	0.3(0–0)	1.234(1.031–1.476) **0.0099**		1.152(0.957–1.387) 0.0872	
tetracyclins DOT	0.1(0–0)	0 (0–0)	1.121 (0.769–1.635) 1.0000		1.058(0.723–1.547) 0.7044	
aminoglycosides DOT	0.1 (0–0)	0 (0–0)	nd^4^ 0.2500		nd^4^ 0.5270	
linezolid DOT	0.3 (0–0)	0.3 (0–0)	1.010 (0.881–1.158) 0.9063		0.980(0.848–1.132) 0.8598	
rifampicin DOT	0.1(0–0)	0.2 (0–0)	0.951 (0.792–1.141) 1.0000		0.912 (0.764–1.089) 0.3286	
nitrofurantoin DOT	0 (0–0)	0 (0–0)	nd^4^ 1.0000		nd^4^ 0.1897	
T/S^3^ DOT	0.2 (0–0)	0.1 (0–0)	0.981(0.848–1.135) 0.2813		0.919 (0.783–1.079) 0.3385	
clindamycin DOT	0.1 (0–0)	0.3 (0–0)	1.107 (0.863–1.420) 0.3672		1.097 (0.844–1.427) 0.4649	

^1^unadjusted p-values (without Bonferroni correction), bold type indicates significant values with p < 0.05

^2^unadjusted p-values (without Bonferroni correction), multivariate analysis of matched pairs with length of stay in hospital as confounder, bold type indicates significant values after Bonferroni correction

^3^ A/S, ampicillin/sulbactam; CEPH, cephalosporin; CI, confidence interval; CVC, central venous catheter; DOT, days of antibiotic therapy; IQR, inter quartil range between the 25% and 75% quartils; LOS, length of stay; OR, odds ratio; P/T, piperacillin/tazobactam; T/S, trimethoprim/sulfamethoxazole

^4^OR and CI not defined because the antibiotic was not used in the control group

Taking into account the length of stay in hospital as possible confounder we performed a secondary analysis of the potential risk factors dialysis, endoscopy, sonography, radiology, and previous antibiotic therapy. The multivariate analysis with confounder revealed only previous antibiotic therapy and previous dialysis as independent risk factors in the strong Bonferroni-adjusted sense. Other potential contact-related risk factors such as radiology, central venous catheter, and endoscopy were not significantly associated with the detection of VRE after taking into account a patient’s length of stay in hospital as confounder (Table 2). Although no single antibiotic was identified as a risk factor after Bonferroni adjustment in the multivariate analysis, the antibiotics meropenem, piperacillin/tazobactam, and vancomycin were associated with the highest risk of in-hospital detection of VRE (Table 2). The results for the sensitivity analyses assuming independent samples are very similar (data not shown).

The most common VRE sequence type among the VRE-positive cases was ST117 (n = 70, 60.3%), followed by ST78 (n = 8, 6.8%), ST203 (n = 7.7%) and ST17 (n = 6. 1%) (Table 3). In all risk groups ST117 was the most frequent MLST-type which accounted for 75.0% of all VRE isolates in the endoscopy group, 63.7% in the radiology group, 60.8% in the antibiotics group, 57.7% in the sonography group and 42.8% in the dialysis group. For the test of association between risk factors and MLST type, the dominant type ST117 was compared to the combined group of all other MLST types. Using this approach only in the dialysis group the frequency of ST117 was significantly lower than in the other risk groups (p = 0.045).

**Table 3:** Distribution of MLST type of VRE-positive surgical inpatients in the risk groups

	VRE isolates, n (%)					
MLST type	all	dialysis	endoscopy	sonography	radiology	antibiotics
17	6 (5,1)	2 (7,1)	3 (9,3)	5 (5,5)	4 (3,9)	6 (5,2)
18	1 (0,8)	1 (3,5)	1 (3,1)	1 (1,1)	1 (0,9)	1 (0,8)
78	8 (6,8)	4 (14,2)	3 (9,3)	6 (6,6)	6 (5,8)	8 (6,9)
80	5 (4,3)	3 (10,7)	1 (3,1)	4 (4,4)	5 (4,9)	5 (4,3)
117	70 (60,3)	12 (42,8)	24 (75)	52 (57,7)	65 (63,7)	70 (60,8)
192	3 (2,5)	0 (0)	0 (0)	3 (3,3)	3 (2,9)	3 (2,6)
202	3 (2,5)	1 (3,5)	0 (0)	3 (3,3)	2 (1,9)	3 (2,6)
203	9 (7,7)	2 (7,1)	0 (0)	6 (6,6)	7 (6,8)	9 (7,8)
233	2 (1,7)	1 (3,5)	0 (0)	2 (2,2)	0 (0)	2 (1,7)
280	1 (0,8)	0 (0)	0 (0)	1 (1,1)	1 (0,9)	1 (0,8)
612	1 (0,8)	0 (0)	0 (0)	1 (1,1)	1 (0,9)	1 (0,8)
769	1 (0,8)	1 (3,5)	0 (0)	1 (1,1)	1 (0,9)	1 (0,8)
780	3 (2,5)	1 (3,5)	0 (0)	3 (3,3)	3 (2,9)	2 (1,7)
889	1 (0,8)	0 (0)	0 (0)	1 (1,1)	1 (0,9)	1 (0,8)
1324	1 (0,8)	0 (0)	0 (0)	1 (1,1)	1 (0,9)	1 (0,8)
nd^1^	1 (0,8)	0 (0)	0 (0)	0 (0)	1 (0,9)	1 (0,8)
total	116	28	32	90	102	115

^1^ST539 or ST948

## Discussion

The present study is a single-centre retrospective matched case-control study covering the period from the second half of 2013 to December 2016, when an increased incidence of VRE-colonised patients was recognized in certain surgical wards. We chose to strictly limit the study period in order to focus on risk factors present in this critical phase. During this surge of VRE the MLST sequence type ST117 was dominant. After taking into account the length of stay in hospital, only previous antibiotic administration (OR 1.069, 95% CI 1.037–1.102) and previous dialysis (OR 3.309, 95% CI 1.387–7.897) were identified as independent risk factors associated with the detection of VRE among surgical inpatients. Our results extend previous data by Kampmeier et al. who reported length of stay on a surgical ICU, long term dialysis and antibiotic treatment with flucloxacillin or piperacillin/tazobactam as the main risk factors für the acquisition of VRE by surgical inpatients on an ICU [13].

Hospitalization preceding the detection of VRE colonisation has been described as an important risk factor for the acquisition of VRE on clinical wards [2, 14–16]. Despite identifying length of stay in hospital as a risk factor, we could not evaluate previous hospitalization because this information was not systematically documented. In our study, the length of stay on an ICU was three times longer in the case group compared to the control group (7.8 vs. 2.6 days), indicating an enhanced risk for selection or acquisition of VRE in this setting.

Admission to an ICU and the duration of the stay are probably also associated with invasive procedures and the need for dialysis, which have been identified as risk factors for the in-hospital acquisition of VRE previously [2, 17]. In our study, the only procedure significantly associated with an enhanced risk for detection of VRE after accounting for length of stay in hospital as a possible confounder was previous dialysis. There was a fourfold increase in the likelihood that a patient in the case group had received some type of dialysis before detection of VRE occurred compared to the control group (OR 3.309, 95% 1.387–7.897 in the analysis adjusted for length of stay). Possible causes for an increased risk of VRE colonisation in patients requiring dialysis could be the frequent contacts with medical staff as well as with medical devices. Another explanation could be the chronic underlying illness of patients requiring dialysis, which may cause repeated infections requiring antibiotic therapies as well as frequent hospital admissions.

Previous antibiotic therapy was associated with an increased risk of VRE colonisation in a number of previous studies [2, 4, 15, 18–22]. The present study clearly corroborates these findings by showing that a previous antibiotic therapy was a significant independent risk factor in the multivariate analysis after taking into account the length of stay as a confounder (OR 1.069, 95% CI 1.037–1.102). In our study the case group patients overall received more than double the amount of antibiotics before the first detection of VRE compared to the control group (p < 0.0001). VRE have a complex resistance mechanism coded by the vancomycin resistance transposon and therefore does not readily develop de novo resistance by point mutations. It is more likely, that previous antibiotic therapy facilitates colonization with VRE or selects VRE already present in low numbers at the time of admission. Both mechanisms could explain the increased risk for detection of VRE in patients exposed to intense or prolonged antibiotic therapy [23–26].

The majority of prescribed antibiotics were beta-lactam antibiotics, especially piperacillin/tazobactam and meropenem, both of which have been associated with an increased risk of in-hospital acquisition of VRE previously [3, 27, 28].

Despite neither DOT with meropenem nor with piperacillin/tazobactam before detection of VRE were significant risk factors after Bonferroni correction, in the exploratory sense both were associated with the highest risk for the detection of VRE. Meropenem and piperacillin/tazobactam both have a broad spectrum of activity, which may result in a selection advantage for resistant enterococci due to reduced competition for nutrients and habitat [3, 20, 29].

We found a difference between both cohorts in previous treatment with second generation cephalosporins which was not significant after Bonferroni correction. Enterococci are naturally resistant to cephalosporins, which could explain the selection of VRE and enterococci in general by this group of antibiotics [3, 27, 30–34].

Fluoroquinolones are suspected of promoting VRE colonisation and infection. [3, 16, 20, 23, 27, 31–33]. In our study, ciprofloxacin was the predominantly prescribed fluoroquinolone. We also found a higher use of ciprofloxacin in the case group compared to the control group. After Bonferroni correction, the difference was not significant and in the exploratory sense the risk was much lower compared to the high-risk antibiotics meropenem and piperacillin/tazobactam.

The role of antibiotic therapy with vancomycin as a risk factor for in-hospital acquisition of VRE has been discussed controversially. On the one hand, there are a number of studies that identified vancomycin as a risk factor for VRE acquisition [2, 3, 16, 27, 30, 33, 35], on the other hand, other studies have found no effect [20, 32, 34]. In our study in the case group, 316 DOT vancomycin before detection of VRE were recorded compared to 61 DOT in the control group. Thus, previous therapy with vancomycin together with meropenem and piperacillin/tazobactam was associated with the highest risk of in-hospital detection of VRE. Another antibiotic class frequently associated with VRE colonisation are the nitroimidazoles with the main representative metronidazole [25, 32, 36]. In our study, increased use of metronidazole was observed in the case group, but the difference was not significant, neither in the strict Bonferroni-adjusted nor in an exploratory sense.

Taken together the current case control study corroborates the numerous previous studies cited above, that associate previous antibiotic therapy with the acquisition of VRE. Despite all this evidence, however, a direct proof that an antibiotic stewardship program prevents the acquisition of VRE in-hospital is missing [37, 38].

The most frequent MLST type in our study was ST117 within clonal complex 17 that after 2010 spread in health care settings in Germany [39]. In our setting the knowledge of the sequence type, however, in most cases was not helpful to track the in-hospital spread of individual VRE isolates, and therefore did not aid in the identification of potential sources of VRE, as this has been described in a recent study using whole genome sequencing for typing of VRE isolates [40]. In the dialysis group ST117 (42.8%) was significantly (p = 0,045) less frequent compared to the frequencies of ST117 in the other risk groups, which was in the range of 57.7–75% of all VRE isolates. With great caution this result could indicate a local spread of non-ST117 VRE types in the central dialysis department. The interpretation of MLST typing results of VRE requires caution because MLST typing of VRE has poor discrimination power and is also limited by frequent gene transfer and recombination events in the dynamic *E. faecium* genome [41]. Because VRE isolates were not archived, it was not possible to reanalyse samples by a more exact molecular analysis, e.g. next generation sequencing which was not available when samples were primarily submitted to the laboratory.

## Limitations

The current study has some important limitations. First, it was not designed as a prospective study based on a VRE screening program for the detection of VRE-positive patients at the time of admission. Therefore, we cannot exclude that upon admissions patients were already colonized with VRE. Even if patients are screened upon admission, a single rectal swab for the detection of VRE is not 100% sensitive and repeated swabs or the use of PCR are required in order to obtain a high degree of certainty that VRE are not already present [42]. Despite we used control plates in order to ensure proper rectal sampling, a single negative swab was sufficient for patients in the control group. Due to the retrospective design of the study we could not further reduce the possibility of false-negative swaps by repeated rectal sampling of patients. As the study was performed retrospectively and room occupancy of patients was not documented in patient files we could not adjust für this risk. Another weakness of our study is that a certain bias in the patient selection cannot be excluded, which is typical for retrospective studies. We attempted to create homogeneity and good comparability of the patients by only including patients from surgical wards and the attached ICU, where the surge of VRE was initially recognized.

## Conclusions

In summary, after taking into account the length of stay in hospital as confounder, with the exception of previous dialysis, the current study identified no directly contact-dependent risk factors for in-hospital detection of VRE. Rather the identification of previous antibiotic therapy as risk factor for VRE suggests that in order to alleviate the presence of VRE on surgical wards and ICUs an antibiotic stewardship program should be implemented that focuses on the identified VRE high-risk antibiotics.

## Data Availability

The datasets used and/or analysed during the current study available from the corresponding author on reasonable request.

## List of Abbreviations

VRE: Vancomycin-resistant *Enterococcus faecium*
ICU: intensive care unit
DOT: days of therapy
OR: odds ratio
